# NAC1 transcriptional activation of LDHA induces hepatitis B virus immune evasion leading to cirrhosis and hepatocellular carcinoma development

**DOI:** 10.1038/s41389-024-00515-4

**Published:** 2024-05-04

**Authors:** Wenbiao Chen, Liliangzi Guo, Huixuan Xu, Yong Dai, Jun Yao, Lisheng Wang

**Affiliations:** 1 Department of Gastroenterology, Shenzhen People’s Hospital, The Second Clinical Medical College, Jinan University; The First Affiliated Hospital, Southern University of Science and Technology, Shenzhen, 518020 China; 2 Clinical Medical Research Center, Guangdong Provincial Engineering Research Center of Autoimmune Disease Precision Medicine, Shenzhen People’s Hospital, The Second Clinical Medical College, Jinan University; The First Affiliated Hospital, Southern University of Science and Technology, Shenzhen, 518020 China

**Keywords:** Cancer, Cell biology

## Abstract

Our study aimed to elucidate the molecular mechanisms underlying NAC1 (nucleus accumbens associated 1) transcriptional regulation of LDHA and its role in HBV immune evasion, thus contributing to the development of cirrhosis and hepatocellular carcinoma (HCC). Utilizing public datasets, we performed differential gene expression and weighted gene co-expression network analysis (WGCNA) on HBV-induced cirrhosis/HCC data. We identified candidate genes by intersecting differentially expressed genes with co-expression modules. We validated these genes using the TCGA database, conducting survival analysis to pinpoint key genes affecting HBV-HCC prognosis. We also employed the TIMER database for immune cell infiltration data and analyzed correlations with identified key genes to uncover potential immune escape pathways. In vitro, we investigated the impact of NAC1 and LDHA on immune cell apoptosis and HBV immune evasion. In vivo, we confirmed these findings using an HBV-induced cirrhosis model. Bioinformatics analysis revealed 676 genes influenced by HBV infection, with 475 genes showing differential expression in HBV-HCC. NAC1 emerged as a key gene, potentially mediating HBV immune escape through LDHA transcriptional regulation. Experimental data demonstrated that NAC1 transcriptionally activates LDHA, promoting immune cell apoptosis and HBV immune evasion. Animal studies confirmed these findings, linking NAC1-mediated LDHA activation to cirrhosis and HCC development. NAC1, highly expressed in HBV-infected liver cells, likely drives HBV immune escape by activating LDHA expression, inhibiting CD8 + T cells, and promoting cirrhosis and HCC development.

## Introduction

Cirrhosis and HCC are two important liver diseases that present major challenges to global public health [1]. Cirrhosis is a chronic liver injury caused by multiple factors, characterized by the remodeling of liver tissue structure and the obstruction of blood flow [2]. The occurrence of cirrhosis significantly amplifies the challenge of treatment due to the limited regenerative potential of liver cells [3]. Moreover, liver cirrhosis is considered one of the primary risk factors for HCC [4]. Treating HCC is highly challenging, and upon diagnosis, most patients experience a brief period of survival [5]. Despite the advancements in medicine, the treatment outcomes for advanced HCC remain unsatisfactory [6]. The disparity in prognoses between the two diseases has imposed significant psychological and financial burdens on patients as well as their families [7]. Infection with the HBV is a crucial factor in the progression of cirrhosis and HCC [8]. Chronic infection with the HBV can result in persistent hepatitis, eventually leading to the development of cirrhosis or HCC [9]. Thus, the prevention and control of HBV infection play a crucial role in reducing the occurrence of liver cirrhosis and HCC [8].

HBV immune escape plays a pivotal role in the development of liver cirrhosis, impeding the ability of the immune system to eliminate the HBV virus, thus leading to persistent infection [10]. This not only heightens the risk of patients developing cirrhosis and liver cancer, but also complicates treatment [11]. Furthermore, advancements in the research on HBV immune evasion enhance our comprehension of the interplay between the virus and the host immune system, yielding significant insights for the formulation of vaccines and antiviral therapies [10]. In conclusion, a comprehensive understanding of the repercussions of cirrhosis and liver cancer, along with the mechanisms behind HBV immune escape, is imperative for the prevention and management of these critical health conditions. Such knowledge can enhance clinical care and support the implementation of preventive measures for patients [9].

Recently, bioinformatics has become increasingly crucial in medical research [12, 13]. Researchers have identified a series of differentially expressed genes (DEGs) through bioinformatics analysis of samples associated with HBV infection-related cirrhosis and HCC [14]. Among them, NAC1 (Nucleic Acid Acyltransferase 1) exhibited significant differential expression [15]. Numerous studies have demonstrated the overexpression of NAC1 in diverse cancer types, suggesting its potential role as a crucial oncogenic factor [16]. Several studies have indicated that the absence of NAC1 in mice enhances the formation of memory CD8 T cells following viral infection. Moreover, NAC1 serves as an inhibitory factor in the development of CD4 T cells, suggesting that targeting NAC1 could be a novel strategy to boost immune memory against pathogens [17, 18]. Subsequent studies have revealed that NAC1 has the ability to regulate the expression of LDHA (lactate dehydrogenase A) either directly or indirectly [19]. LDHA is a critical enzyme involved in lactate fermentation, which is tightly linked to the metabolic reprogramming of tumor cells [20]. Elevated LDHA activity promotes the proliferation and invasiveness of cancer cells [21]. Therefore, NAC1 may have a significant impact on the advancement of HCC through the regulation of LDHA [16]. This discovery not only offers a fresh perspective on the pathogenesis of HCC, but also provides novel insights for targeted therapies against NAC1 and LDHA [22].

The purpose of this study is to explore the expression of NAC1 in liver cells infected with HBV and its potential biological functions. In conclusion, researchers are committed to investigating the activation of LDHA transcription by NAC1 and its impact on the immune response, particularly in the suppression of CD8 + T cells, which ultimately leads to the induction of mechanisms for HBV immune evasion. This study is significant because it provides a comprehensive understanding of the occurrence of liver cirrhosis and HCC following HBV infection. Additionally, it introduces new targets for future therapeutic strategies against these diseases.

## Results

### Bioinformatics analysis was used to screen out differentially expressed genes in HBV-infected liver cirrhosis/HCC samples

HCC is the major malignant tumor of liver tissue in patients with cirrhosis, and patients with cirrhosis still face the challenges of high postoperative recurrence rate and low survival rate [23, 24]. However, HBV remains the most significant risk factor for its occurrence. To identify key genes related to the impact of HBV on liver cirrhosis and HCC development, we obtained two chip datasets, GSE36376 and GSE87630, related to HBV infection, liver cirrhosis and HCC from the GEO database.

**Fig. 1:**
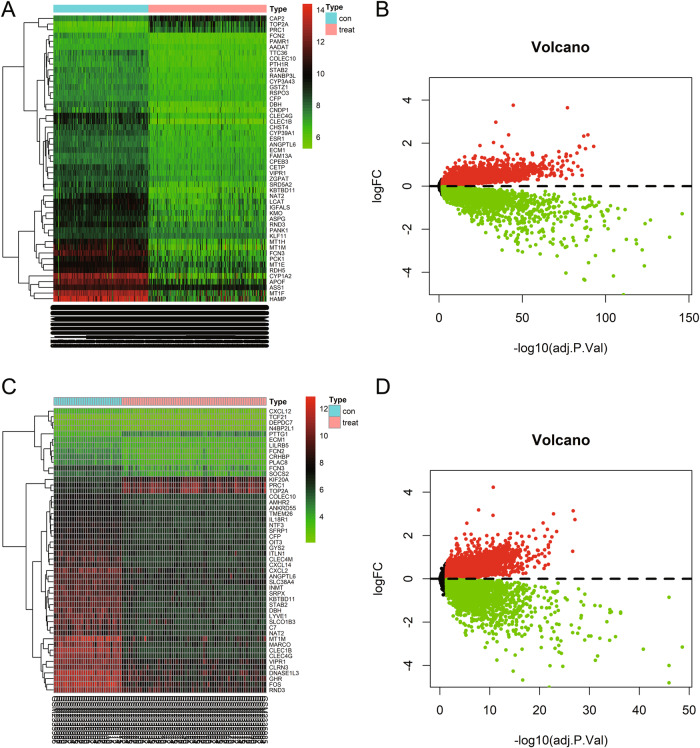
Selection of differentially expressed genes in HBV infection-related cirrhosis/HCC samples based on GEO chip dataset. Note: **A** Heat map of differentially expressed genes between the normal control group (Normal, *n* = 193) and the HBV-related HCC group (HBV-HCC, n = 240) in the chip dataset GSE36376, with gene clustering performed based on gene expression levels. The histogram in the upper right corner represents the color scale, with red indicating upregulated genes and green indicating downregulated genes. **B** Volcano plot of differentially expressed genes between the normal control group and the HBV-related HCC group in the chip dataset GSE36376, with green dots representing downregulated genes, red dots representing upregulated genes, and black dots representing no significant difference between the groups. **C** Heat map of differentially expressed genes between the normal control group (Normal, *n* = 30) and the HBV-related HCC group (HBV-HCC, *n* = 64) in the chip dataset GSE87630. **D** Volcano plot of differentially expressed genes between the normal control group and the HBV-related HCC group in the chip dataset GSE87630.

The analysis of differential gene expression results shows that a total of 10776 differentially expressed genes were screened from the dataset GSE36376, including 5161 upregulated genes and 5615 downregulated genes (Fig. 1A, B); 7659 differentially expressed genes were screened from dataset GSE87630, including 4649 upregulated genes and 3010 downregulated genes (Fig. 1C, D).

### Multi-database joint analysis and screening of prognostic-related genes affecting liver cirrhosis and HCC occurrence in HBV infection

**Fig. 2:**
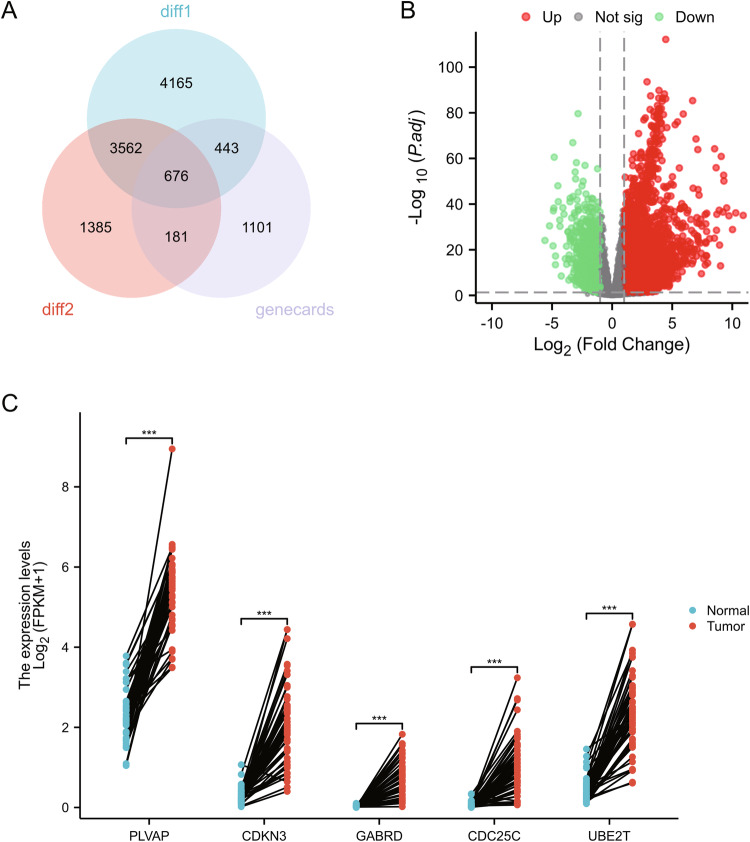
Screening of prognostic genes related to HBV infection-related liver cirrhosis/HCC based on GEO and TCGA databases. Note: **A** The Venn diagram of the intersection between the DEGs of chip datasets GSE36376 and GSE87630 and the GeneCards database; **B** A volcano plot of differentially expressed genes between the normal control group and the HCC group in the TCGA-LIHC dataset; **C** Results of differential analysis of the top 5 HCC-related genes based on TCGA-LIHC data in paired samples (*** represents *P* < 0.001 in gene expression comparison between paired tumor and normal samples).

To identify tumor-associated genes related to the occurrence and development of HCC secondary to liver cirrhosis, we first used a Venn diagram to intersect the disease-related genes in the GEO database chip DEGs and GeneCards database. There are a total of 676 intersecting genes between the DEGs of GES36376 and GSE87630 and the relevant genes for “Liver cancer” in the GeneCards database (Fig. 2A). These genes may be closely related to the occurrence of liver cirrhosis and HCC associated with HBV infection.

We further performed differential expression and prognosis correlation screening on this subset of related genes based on the TCGA database. In the TCGA-LIHC dataset, 475 genes showed differential expression between normal and tumor samples (Fig. 2B), and the differential results of the top 5 significantly different genes in paired samples are shown in Fig. 2C.

### NAC1 may be a key gene that affects liver cirrhosis and HCC occurrence and development after HBV infection

**Fig. 3:**
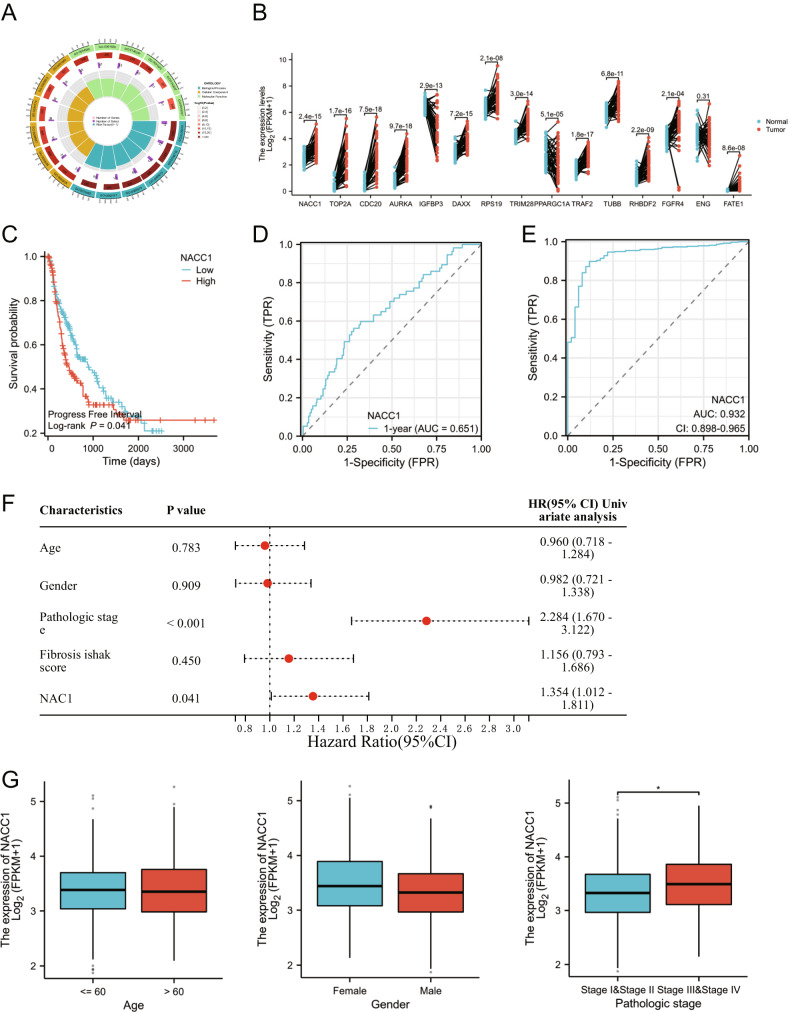
Identification of key prognostic genes through Gene Ontology (GO) functional enrichment analysis and screening of The Cancer Genome Atlas Liver HCC (TCGA-LIHC) dataset. Note: **A** A circle plot of GO enrichment analysis for 676 HBV infection-related intersection genes in liver cirrhosis and HCC (from outside to inside, showing pathway number, gene number in pathway, number and proportion of differentially enriched genes); **B** Pairwise differential analysis of 15 key genes based on TCGA-LIHC dataset; **C** Survival curve of NAC1 (red: high expression group, blue: low expression group); **D**, **E** ROC curve of NAC1 for predicting patient survival and disease diagnosis (the area under the curve (AUC) represents the predictive accuracy); **F** Independent prognostic analysis forest plot based on the TCGA-LIHC dataset; **G** Correlation analysis between NAC1 expression and various clinical factors; *represents *P* < 0.05, **0.001 < *P* < 0.01, ***P < 0.001.

To further screen the key genes that influence HBV infection-related liver cirrhosis/HCC occurrence and development, we performed GO functional enrichment analysis on the aforementioned 676 intersection genes. The enrichment analysis results of GO are divided into three categories: BP (Biological Process), CC (Cellular Component) and MF (Molecular Function), as shown in Fig. 3A. To identify the pathway genes associated with HBV infection and HCC, we focused on the gene enrichment of the pathway related to molecular functional (MF) categories. The results showed that three pathways, “ubiquitin protein ligase binding”, “growth factor binding” and “histone deacetylase binding”, were most closely related to HBV infection and HCC occurrence, based on the comprehensive function-enriched *p*-value, intersection gene set proportion, and molecular pathways reported in previous studies related to HBV infection.

We extracted 75 enriched genes from 3 pathways and intersecting with 169 prognostic-related genes from TCGA database resulted in 15 genes closely associated with the HBV infection-induced liver cirrhosis and HCC, namely TOP2A, CDC20, AURKA, IGFBP3, DAXX, RPS19, TRIM28, PPARGC1A, TRAF2, TUBB, RHBDF2, FGFR4, ENG, NAC1, and FATE1. Based on the differential expression analysis of paired samples from the TCGA database (Fig. 3B), TOP2A, CDC20, AURKA, TRAF2, and NAC1 were found to be the most significantly differentially expressed genes. In addition, literature related to the above-mentioned prognostic genes and HCC was screened through the PubMed database, and the results showed that NAC1 (nucleus accumbens associated 1, also known as NACC1) is closely associated with the occurrence and development of HCC [25–27]. However, the research on its molecular mechanism is still very limited at present.

Subsequently, we further validated the relationship between NAC1 and HCC based on the TCGA database. Survival analysis results showed that patients with high expression levels of NAC1 had significantly lower survival rates (Fig. 3C). Subsequently, we evaluated the accuracy of NAC1 in predicting patient prognosis using the ROC curve. The results showed an accuracy rate of 0.65 when predicting patient survival time (Fig. 3D). However, if used only to diagnose the disease, the accuracy rate could reach 90% (Fig. 3E). Further independent prognostic analysis was performed to determine if NAC1 could serve as an independent prognostic factor in patients. The results showed that the prognostic *P* value of NAC1 was 0.041, and the HR (95% CI) was 1.354 (1.012–1.811), indicating that NAC1, as a risk factor for HCC, could act as a prognostic factor for patients independently of other factors (Fig. 3F). Finally, we also analyzed the correlation between NAC1 and other clinical factors, among which NAC1 showed significant correlation with tumor staging, while no significant correlation was observed with other factors (Fig. 3G).

### GO and KEGG functional analysis predicted that NAC1 may be involved in the HBV immune escape process

**Fig. 4:**
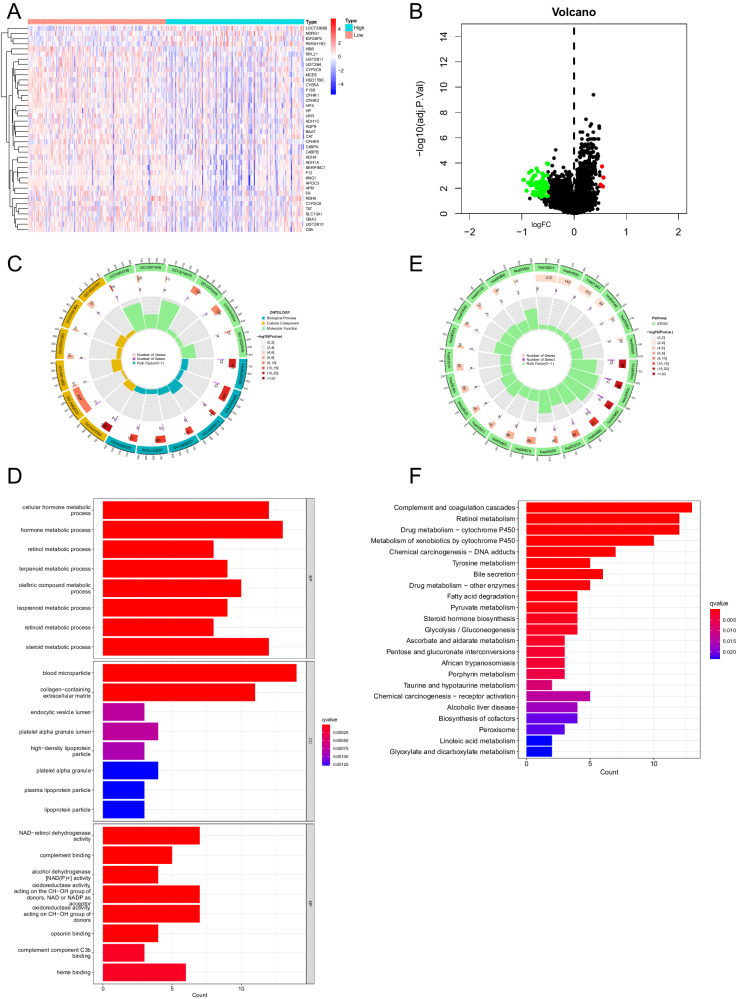
Functional analysis of genes related to NAC1 in GEO database chip. Note: **A** Heat map of differentially expressed genes (DEGs) in the merged datasets of chip GES36376 and GSE87630 grouped by NAC1 expression levels; **B** Volcano plot of DEGs in the merged datasets of chip GES36376 and GSE87630; **C**, **D** Circular and bar charts of Gene Ontology (GO) functional enrichment analysis of DEGs (where size and color of the bar chart represent the number and significance of enriched genes in the pathway); **E**, **F** Circular and bar charts of Kyoto Encyclopedia of Genes and Genomes (KEGG) functional enrichment analysis of DEGs.

In order to investigate the key downstream factors or signaling pathways that NAC1 participates in the development of liver cirrhosis and HCC, we merged the GEO datasets GES36376 and GSE87630, and performed differential expression analysis after grouping based on NAC1 expression levels. The analysis of differential gene expression revealed 64 differentially expressed genes, including 4 up-regulated genes and 60 down-regulated genes, which were selected by the Communist Party (Fig. 4A, B). Subsequently, Gene Ontology (GO) and Kyoto Encyclopedia of Genes and Genomes (KEGG) pathway enrichment analysis were performed on these differentially expressed genes.

The results of GO enrichment analysis on the BP level in GO functional analysis showed that these genes were mainly enriched in signal pathways such as “cellular hormone metabolic process”, “hormone metabolic process” and “humoral immune response” (Fig. 4C, D); KEGG pathway enrichment analysis showed that differentially expressed genes were mainly enriched in signal pathways such as “Complement and coagulation cascades”, “Retinol metabolism” and “Chemical carcinogenesis - DNA adducts” (Fig. 4E, F). The above results indicate that NAC1 is involved in the related genes of HCC, mainly in the liver metabolic functions of bile secretion and metabolism, substances such as drugs and alcohol metabolism, as well as immune response processes. Further literature review revealed that overexpression of NAC1 in tumors can promote the progression of melanoma through LDHA-mediated immune escape [16]. Therefore, we speculate that NAC1 mediates its involvement in HBV immune escape, promoting the occurrence and development of liver cirrhosis and HCC by transcriptionally regulating LDHA expression.

### NAC1 transcriptionally activates LDHA to promote immune cell apoptosis and induce HBV immune escape

**Fig. 5:**
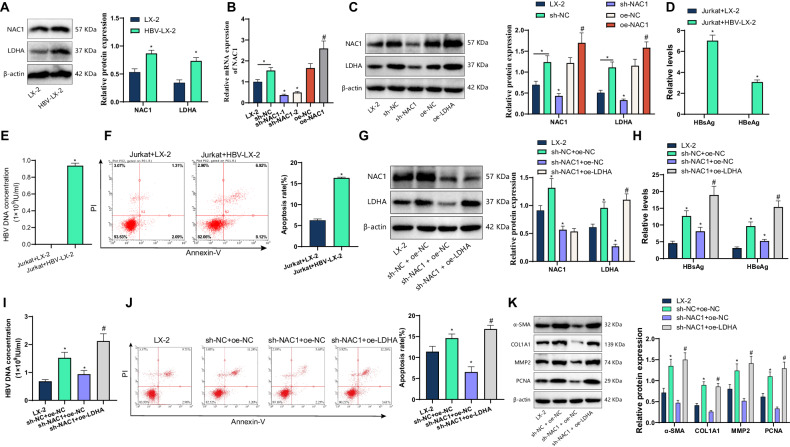
NAC1 transcriptionally regulates LDHA expression to mediate HBV immune escape. Note: **A** Western Blot was used to detect the expression of NAC1 and LDHA in human hepatic stellate cells (LX-2) after HBV infection; **B** RT-qPCR was used to detect the efficiency of overexpression and silencing of NAC1 in HBV-LX-2 cells; **C** Western Blot was used to detect the expression of NAC1 and LDHA in HBV-LX-2 cells; **D** HBsAg, HBeAg ELISA kit was used to detect the level of HBsAg and HBeAg in the culture supernatant; **E** ELISA kit was used to detect the level of HBV DNA in the culture supernatant; **F** Flow cytometry was used to detect the apoptosis rate of immune cells Jurkat; **G** Western Blot was used to detect the expression of NAC1 and LDHA in HBV-LX-2 cells; **H** HBsAg, HBeAg ELISA kit was used to detect the level of HBsAg and HBeAg in the culture supernatant; **I** ELISA kit was used to detect the level of HBV DNA in the culture supernatant; **J** Flow cytometry was used to detect the apoptosis rate of immune cells Jurkat; **K** Western Blot was used to detect the expression of α-SMA, COL1A1, MMP2, and PCNA in LX-2 cells infected with HBV; *denotes *P* < 0.05 compared with the LX-2 group or sh-NC group or Jurkat + LX-2 group or sh-NC + oe-NC group, # denotes *P* < 0.05 compared with the oe-NC group or sh-NAC1 + oe-NC group; all cell experiments were independently repeated three times.

By Western Blot analysis of NAC1 and LDHA expression in human hepatic stellate cells (LX-2) after HBV infection, it was found that compared to LX-2 cells, there was a significant increase in the protein expression of NAC1 and LDHA in LX-2 cells after HBV infection (Fig. 5A). Overexpression or silencing of NAC1 was conducted in HBV-infected LX-2 cells. RT-qPCR results showed successful silencing or overexpression of NAC1. Among them, sh-NAC1-1 had the best silencing effect and can be used for subsequent experiments (Fig. 5B). In addition, Western Blot analysis revealed that overexpression of NAC1 enhances LDHA expression, while silencing NAC1 suppresses LDHA expression (Fig. 5C).

We co-cultured HBV-infected or uninfected LX-2 cells with immune cells Jurkat. ELISA and flow cytometry detection results showed that compared with the Jurkat + LX-2 co-culture group, the HBV-LX-2 group had significantly increased levels of HBsAg, HBeAg, and HBV DNA in the cell culture supernatant, and the apoptosis rate of Jurkat immune cells was significantly increased (Fig. 5D–F).

To investigate the mechanism of NAC1-mediated LDHA expression in HBV immune escape, we silenced NAC1 or overexpressed LDHA in HBV-LX-2 cells. Western blot analysis revealed that, compared to the sh-NC+oe-NC group, the expression levels of NAC1 and LDHA proteins were significantly decreased in HBV-LX-2 cells in the sh-NAC1+oe-NC group. Moreover, in comparison to the sh-NAC1+oe-NC group, the expression of NAC1 protein remained unchanged whereas the expression of LDHA protein was significantly increased in HBV-LX-2 cells in the sh-NAC1+oe-LDHA group (Fig. 5G).

In addition, it was found that the HBsAg, HBeAg, and HBV DNA contents in the cell supernatant co-cultured by Jurkat cells and HBV-LX-2 cells with silenced NAC1 significantly decreased, and the apoptosis rate of Jurkat immune cells decreased significantly. However, compared with the single knockdown of NAC1, the HBsAg, HBeAg, and HBV DNA contents in the cell supernatant co-cultured by HepAD38 cells with silenced NAC1 and overexpressed LDHA significantly increased, and the apoptosis rate of Jurkat immune cells increased significantly (Fig. 5H–J). The Western blot test found that compared with the sh-NC + oe-NC group, the α-SMA, COL1A1, MMP2, and PCNA proteins decreased in HBV-LX-2 cells co-cultured with Jurkat cells in the sh-NAC1 + oe-NC group. Compared with the sh-NAC1 + oe-NC group, the α-SMA, COL1A1, MMP2, and PCNA proteins increased in HBV-LX-2 cells co-cultured with Jurkat cells in the sh-NAC1 + oe-LDHA group (Fig. 5K).

### CD137 monoclonal antibody can induce liver fibrosis and HCC in HBV transgenic mice

**Fig. 6:**
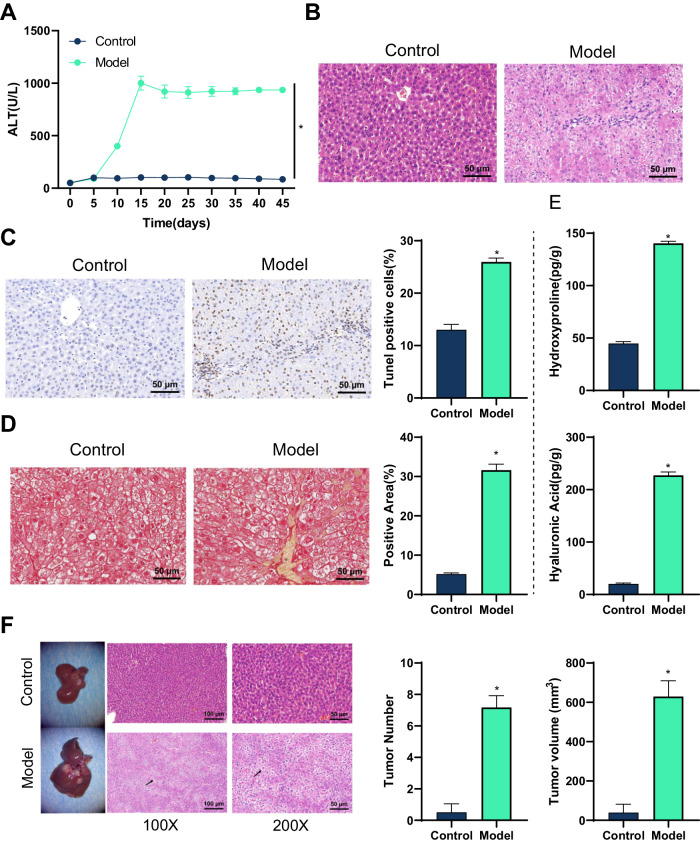
CD137 monoclonal antibody induces liver cirrhosis and HCC in HBV transgenic mice. Note: **A** Detection of the release of alanine transaminase (ALT) in mouse serum; **B** Observation of histopathological changes in mouse liver by H&E staining; **C** Observation of hepatocyte apoptosis in mouse liver tissue by TUNNEL staining; **D** Analysis of collagen deposition/fibrosis in mouse liver by Sirius Red staining; **E** Detection of the levels of hydroxyproline and hyaluronic acid, liver fibrosis factors, in mouse liver tissue homogenates by ELISA; **F** Observation of nodules in mouse liver and histopathological changes by H&E staining, and black arrows indicate nodules. *indicates a significant difference compared to the Control group, *P* < 0.05, *N* = 6.

The initial liver inflammation induced by CD137 monoclonal antibody is due to the activation of memory-like non-specific CD8 + T cells, and sustained CD137 stimulation may be a liver immunopathological factor contributing to chronic HBV infection [28]. Inject HBV transgenic mice intraperitoneally with 100 µg CD137 antibody or control agent once a week for seven weeks. Regularly collect serum to monitor the release of serum alanine aminotransferase (ALT). As shown in Fig. 6A, ALT levels in HBV transgenic mice started to rapidly increase from day 10 after the first injection and remained high throughout the entire injection process. H&E staining showed diffuse necrotizing inflammation in liver tissue sections of Model group mice, with visible mononuclear cell infiltration and deep infiltration of liver cells (Fig. 6B). TUNEL staining showed a significant increase in hepatocyte apoptosis (Fig. 6C).

Sirius Red staining was used to analyze liver collagen deposition/fibrosis. CD137-induced HBV transgenic mice exhibited severe fibrosis (Fig. 6D). Furthermore, CD137 induction significantly increased the levels of hepatic fibrosis factors hydroxyproline and hyaluronic acid in the liver tissue homogenates of HBV transgenic mice (Fig. 6E). These results indicate that CD137 monoclonal antibody successfully induces liver damage and inflammation in HBV transgenic mice.

After 7 injections (at 6 months), we observed the occurrence of large liver tumor nodules (≥3 mm^2^) in the Model group mice, while only 2 control group mice had 1 or 2 smaller nodules (≤2 mm^2^). Histopathological examination of the liver tissues stained with H&E revealed typical liver morphology. The total number and volume of liver tumor nodules in the Model group mice showed a significant increase (Fig. 6F).

Interesting results indicate that CD137 monoclonal antibody can induce persistent liver inflammation in HBV transgenic mice, causing severe liver damage, leading to fibrosis, cirrhosis, and HCC.

### Animal experiments in vivo have confirmed that NAC1 transcriptional activation induces LDHA to induce HBV immune escape, thereby promoting liver cirrhosis and HCC occurrence

**Fig. 7:**
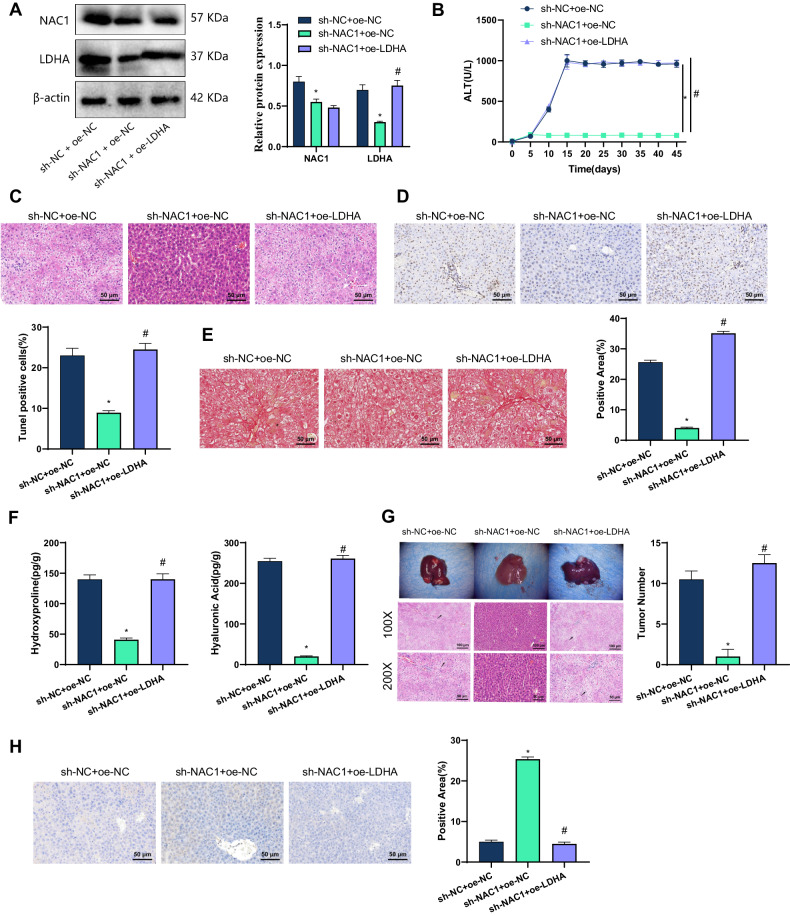
In vivo experimental validation of NAC1-mediated transcriptional activation of LDHA inducing HBV immune escape involved in the development of liver cirrhosis and HCC. **A** Western blot analysis was performed to detect the protein expression of NAC1 and LDHA in mouse liver tissue; **B** ALT release in mouse serum was measured; **C** H&E staining was used to observe pathological changes in mouse liver; **D** TUNNEL staining was used to investigate hepatocyte apoptosis in mouse liver tissue; **E** Sirius Red staining was used to analyze collagen deposition/fibrosis in mouse liver; **F** ELISA was used to measure the levels of hepatic fibrosis factors hydroxyproline and hyaluronic acid in mouse liver tissue homogenates; **G** Liver morphology and H&E staining were used to observe the occurrence of liver nodules in mice, with black arrows indicating nodules; **H** IHC was used to examine the expression of CD8 protein in mouse liver tissue. * indicates *P* < 0.05 compared with the sh-NC + oe-NC group, and # indicates *P* < 0.05 compared with the sh-NAC1 + oe-NC group, *N* = 6.

To further explore the molecular mechanisms underlying the involvement of NAC1-mediated LDHA expression in HBV immune escape, liver cirrhosis, and HCC development, we treated HBV transgenic mice induced by CD137 monoclonal antibodies with NAC1 silencing, or simultaneous NAC1 silencing and overexpression of LDHA. The results of Western Blot showed that compared to the sh-NC + oe-NC group, the levels of NAC1 and LDHA proteins in mouse liver tissue of the sh-NAC1 + oe-NC group were significantly reduced. Compared to the sh-NAC1 + oe-NC group, there was no significant difference in NAC1 protein level but LDHA protein level was significantly increased in mouse liver tissue of the sh-NAC1 + oe-LDHA group (Fig. 7A).

In addition, compared with sh-NC + oe-NC group, the content of ALT in the liver tissue of HBV transgenic mice in sh-NAC1 + oe-NC group was significantly reduced, and the inflammation and infiltration of liver tissue were relieved, while the apoptosis of liver cells was significantly decreased. Compared with sh-NAC1 + oe-NC group, the content of ALT in the liver tissue of HBV transgenic mice in sh-NAC1 + oe-LDHA group was significantly increased, and diffuse necrotic inflammation and mononuclear cell infiltration were visible in the liver tissue, while the apoptosis of liver cells was significantly increased (Fig. 7B–D). Sirius Red staining analysis showed that compared with sh-NC + oe-NC group, collagen deposition/fibrosis in liver tissue of HBV transgenic mice in sh-NAC1 + oe-NC group was significantly reduced; compared with sh-NAC1 + oe-NC group, collagen deposition/fibrosis in liver tissue of HBV transgenic mice in sh-NAC1 + oe-LDHA group was significantly increased (Fig. 7E). ELISA test results showed that compared with the sh-NC + oe-NC group, the level of hepatic fibrosis factors hydroxyproline and hyaluronic acid in the liver tissue homogenate of HBV transgenic mice in the sh-NAC1 + oe-NC group was significantly decreased; compared with the sh-NAC1 + oe-NC group, the level of hepatic fibrosis factors hydroxyproline and hyaluronic acid in the liver tissue homogenate of HBV transgenic mice in the sh-NAC1 + oe-LDHA group was significantly increased (Fig. 7F).

After 7 injections (at 6 months), the nodules in the mouse liver tissue were observed by H&E staining. Compared with sh-NC + oe-NC group, sh-NAC1 + oe-NC group showed a significant reduction in the maximum tumor volume and tumor nodules in the liver tissue of HBV transgenic mice; compared with sh-NAC1 + oe-NC group, sh-NAC1 + oe-LDHA group showed a significant increase in the maximum tumor volume and tumor nodules in the liver tissue of HBV transgenic mice (Fig. 7G). Immunohistochemical analysis found that compared to sh-NC + oe-NC group, the number of CD8 + T cells in the liver tissues of HBV transgenic mice in sh-NAC1 + oe-NC group was increased; and compared to sh-NAC1 + oe-NC group, the number of CD8 + T cells in the liver tissues of HBV transgenic mice in sh-NAC1 + oe-LDHA group was decreased (Fig. 7H).

## Discussion

Immune escape following infection with HBV is a critical factor in the development of liver cirrhosis and HCC [29]. Immune escape refers to the pathogens’ ability, such as HBV, to evade or diminish attacks from the host immune system, enabling them to persist within the host’s body [9]. Persistent HBV infection and evasion of the immune response can result in chronic liver inflammation, which can slowly progress to cirrhosis and potentially promote the development of HCC [30]. In this study, NAC1 plays a significant role in this process [19]. NAC1 can activate LDHA transcriptionally, exerting further influence on cell energy metabolism and proliferation. This leads to the creation of a favorable survival environment for HBV [16]. In addition, NAC1 enhances the immune evasion mechanism of HBV by suppressing CD8 + T cells, which play a crucial role in the cytotoxic immune response [16]. This study offers a novel mechanistic perspective to comprehend the development of HBV-associated liver cirrhosis and liver cancer [31].

NAC1, a crucial molecular regulatory factor, has shown significance in diverse cancers, specifically in the proliferation, invasion, and migration of cancer cells [32]. Abnormal expression of NAC1 is strongly correlated with the progression of diseases and unfavorable prognosis in other types of cancer [16]. However, recent studies have unveiled the distinctive expression pattern and mechanism of action of NAC1 in liver cells infected with HBV [33]. NAC1 demonstrates notable differential expression, particularly in the context of HBV infection [34]. Importantly, NAC1 may facilitate the advancement of liver cirrhosis and HCC by activating LDHA transcription [16]. The activation of LDHA has been considered a crucial step in cancer metabolic reprogramming, promoting cancer growth and progression [35]. Hence, NAC1 plays a pivotal role in both HBV-infected liver cells, making it a potential therapeutic target for liver cirrhosis and HCC [16].

This study employed bioinformatics methods to provide initial evidence suggesting that NAC1 may regulate LDHA expression through transcriptional regulation. This regulatory mechanism potentially contributes to HBV immune escape, thus promoting the development of liver cirrhosis and HCC [36]. Understanding the accuracy and reliability of this mechanism is crucial for ensuring the credibility of scientific research [37]. Firstly, it is important to highlight the reliability and accuracy of the bioinformatics methods employed. This ensures that the identified differentially expressed genes and key signaling pathways are dependable and will aid in subsequent experimental validation [38]. In this study, both in vitro cell experiments and in vivo animal experiments were essential [39]. We investigated the impact of NAC1 transcription activation on apoptosis in immune cells and its role in inducing immune evasion of HBV through in vitro cell experiments [35]. The conducted experiments offer direct evidence that substantiates the initial conclusions drawn from the analysis of bioinformatics, while also unveiling potential molecular mechanisms [40]. Moreover, in vivo animal experiments provided further confirmation regarding the involvement of NAC1 in activating LDHA transcription. Furthermore, these experiments demonstrated how NAC1 induces immune escape from HBV, thereby promoting the development of liver cirrhosis and HCC [16]. Validation is a critical component for assessing the credibility of research findings, providing us with greater confidence in the vital role of NAC1 in this process [41]. In conclusion, this study presents robust scientific evidence to clarify the role of NAC1 in immune evasion by HBV through the integration of bioinformatics techniques and experimental validation [16]. The reliability and accuracy of validation not only contribute to a better understanding of this mechanism, but also provide robust support for the development of future disease treatments [38].

**Fig. 8:**
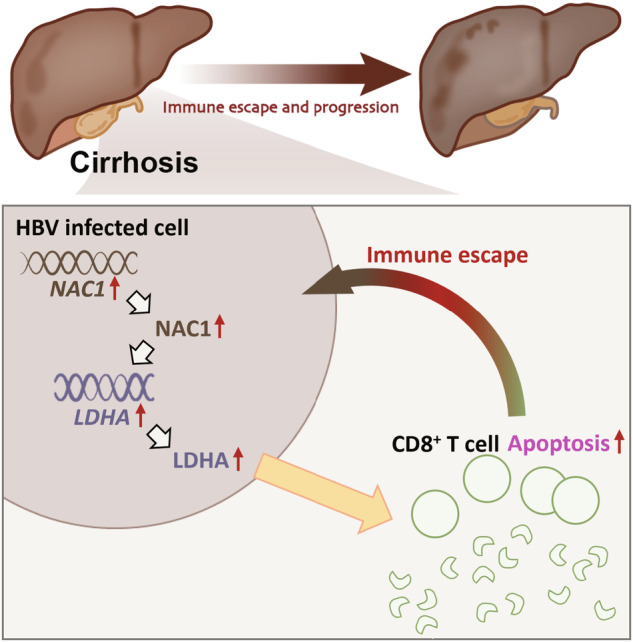
Molecular mechanisms of NAC1-mediated transcriptional regulation of LDHA involved in HBV immune escape contributing to the development of liver cirrhosis and HCC. Note: NAC1 activates LDHA expression through transcription and suppresses CD8 + T cells, thus inducing HBV immune escape and ultimately promoting the occurrence of liver cirrhosis and HCC.

This study’s conclusion highlights the significant role of NAC1 in HBV-infected hepatocytes (Fig. 8). NAC1 mediates HBV immune evasion by transcriptionally activating LDHA, ultimately resulting in the development of liver cirrhosis and HCC. This discovery is highly significant for the treatment of cirrhosis and HCC, as it introduces new therapeutic targets and strategies. Targeting NAC1 and LDHA interventions have the potential to serve as future therapeutic approaches for these diseases. Nevertheless, it is essential to validate the results of this study through clinical practice in order to ascertain the applicability of these findings in the human body. Additional research in clinical studies is necessary to confirm whether NAC1 and LDHA can effectively serve as therapeutic targets, offering improved treatment options for patients with HBV-related liver cirrhosis and HCC. Moreover, future studies should further investigate alternative molecular signaling pathways and different types of immune cells to acquire a more comprehensive comprehension of the pathogenesis of diseases associated with HBV. This will contribute to the development of innovative treatment methods, offer patients additional treatment options, and enhance their survival rate as well as their quality of life. In conclusion, this study has identified new research avenues for the treatment of HBV-related liver cirrhosis and HCC, emphasizing the importance and potential significance for future exploration.

## Materials and methods

### Transcriptome sequencing chip data acquisition

With normal samples as controls, the chip datasets GSE36376 and GSE87630 were analyzed for differential gene expression using the “limma” package in R software, and the false discovery rate (FDR) method was used to correct the differential *P* values. Differentially expressed genes (DEGs) were obtained by screening with a significance level of FDR < 0.05 as the threshold for differential gene selection. The obtained DEGs were visualized using the “pheatmap” package in R for generating heatmap and volcano plot of expression data.

### Intersectional gene screening

The genes associated with “HCC” were acquired from the GeneCards database. Subsequently, we utilized the “VennDiagram” package within the R software to identify the intersection between the differentially expressed genes found in the chip datasets GSE36376 and GSE87630 and the genes associated with HCC from the GeneCards database. These overlapping genes were then designated as candidate genes for subsequent analysis [42].

### Prognostic analysis

Based on the FPKM format RNAseq data from LIHC project in TCGA database (https://portal.gdc.cancer.gov/), R software was used to perform differential expression analysis and prognosis-related gene selection on the intersection genes, with prognostic parameter setting type as Progress Free Interval (PFI). Subsequently, differential expression analysis of paired samples was performed on the top 5 genes with significant differences. Survival analysis, ROC curve visualization, independent prognostic analysis, and clinical correlation analysis were carried out using the “survminer” package (version 0.4.9) and the “survival” package (version 3.2–10) in R software, citing relevant literature. During the analysis, grouping was performed based on gene expression levels of 0–50% and 50–100%.

### Enrichment analysis of functionalities

The chip datasets, namely GSE36376 and GSE87630, were subjected to identical preprocessing steps involving gene screening and data integration, a process executed using the “limma” package within the R software. Following the exclusion of normal samples, a differential expression analysis was conducted. For the subsequent functional annotation and pathway analysis, we harnessed the “ClusterProfiler” package within the R software. The Gene Ontology (GO) enrichment analysis encompassed three hierarchical levels: Biological Process (BP), Cellular Component (CC), and Molecular Function (MF), with a significance threshold set at *P* < 0.05. Additionally, Kyoto Encyclopedia of Genes and Genomes (KEGG) enrichment analysis was executed to identify cellular functions and signaling pathways primarily influenced by the differentially expressed genes, employing a notable enrichment cutoff of *P* < 0.05.

### Cell culture and transfection

Human normal hepatic stellate cells LX-2 were purchased from ATCC (https://www.atcc.org/) and cultured in DMEM (Gibco, 11965092, USA) containing 10% fetal bovine serum (FBS) at 37°C in a 5% CO2 environment. LX-2 cells were infected with HBV at 1012 U/L for 36 h [43].

Following HBV infection, LX-2 cells were categorized into distinct experimental groups based on various treatments: the sh-NC group (silencing lentivirus control group), sh-NAC1-1 group (silenced NAC1 slow virus group 1), sh-NAC1-2 group (silenced NAC1 slow virus group 2), oe-NC group (overexpression slow virus control group), oe-NAC1 group (overexpression slow virus NAC1 group), sh-NC + oe-NC group (silenced slow virus control + overexpression slow virus control group), sh-NAC1 + oe-NC group (silenced NAC1 slow virus + overexpression slow virus control group), and sh-NAC1 + oe-LDHA group (silenced NAC1 slow virus + overexpression slow virus LDHA group). The specific knockdown sequences are detailed in Table S1. Both the silencing and overexpression of NAC1 are mediated by lentivirus. The recombinant lentiviruses for gene silencing and overexpression were prepared and supplied by Shanghai Sangon Biotech Co., Ltd. (Shanghai, China). Cell transfection was carried out as follows: LX-2 cells in the logarithmic growth phase were harvested and resuspended at a concentration of 5 × 10^4^ cells/mL. Subsequently, 2 mL of this cell suspension was seeded into each well of a 6-well plate, followed by an overnight incubation at 37 °C. In each well, recombinant lentiviruses at a concentration of 1 × 10^8^ TU/mL, either silencing or overexpressing the target gene, were added. After 24 h of infection, the efficiency of GFP infection was assessed under a fluorescence microscope, and cells displaying robust effects were selected for subsequent experiments. Each experimental procedure was replicated three times for accuracy and consistency.

### Co-cultivation system

HBV-infected or uninfected LX-2 cells were co-cultured with immune cells Jurkat. Jurkat cells were placed in the upper chamber of Transwell while LX-2 cells were placed in the lower chamber. This process was done to detect the impact of LX-2 cells on the apoptosis and proliferation of immune cells Jurkat [10].

### ELISA detection

The assessment of HBV-DNA (CS11321, Shanghai ChunShi Biological Technology Co., Ltd.) and HBeAg (CR-018, Lantu Biotech., China) expression levels in the culture medium’s supernatant was performed employing an ELISA kit. In this procedure, both the sample and control samples were introduced into separate wells. Subsequently, an Ab-HRP complex was added and the mixture was incubated at 37°C for one hour, followed by five washes with PBST. Following the washing steps, 100 mL of substrate solution was added to each well and incubated for 15 min, after which the reaction was halted. The absorbance at 450 nm was quantified utilizing a microplate reader from BIO-RAD, USA [43].

### Flow cytometry

To assess cell apoptosis, flow cytometry was employed as the detection method. Initially, the cells were washed three times with cold PBS. Subsequently, apoptosis detection was carried out using the Annexin V-FITC/PI apoptosis detection kit sourced from BD Pharmingen, USA. The cells were resuspended in 500 μL of binding buffer, and under conditions devoid of light, 5 μL of Annexin V-FITC and 5 μL of PI were added. The mixture was thoroughly mixed and then incubated for a duration of 15 min. Following this incubation period, cell apoptosis was quantified using a BD FACSCalibur flow cytometer [44].

### Construction of a mouse model of liver cirrhosis

The C57BL/6-HBV transgenic mice were purchased from Beijing Weitongda Biotechnology Co., Ltd. (Beijing, China). The mice were raised under specific pathogen-free conditions at the animal facility of the Institute of Biophysics, Chinese Academy of Sciences, and all animal-related research was approved by the Institutional Animal Care and Use Committee (No. SZPH-AE-21018).

In this experimental study, mice were subjected to various treatments. The modeling group received Mouse CD137 monoclonal antibody (Clone 2 A, SRP0607, Sigma-Aldrich), while Mouse IgG (12-370, Sigma-Aldrich) served as the control. The CD137 antibody (Clone 2 A) was administered via intraperitoneal injection at a dose of 100 µg per week for seven consecutive weeks. Serum collection and monitoring of serum ALT levels were carried out regularly throughout the study. The mice were categorized into five distinct groups, each consisting of 12 mice. These groups were as follows: Control (mice treated with IgG), Model group (mice treated with CD137 monoclonal antibody Clone 2 A), sh-NC + oe-NC group (mice initially treated with control lentivirus and overexpression lentivirus for 3 days, followed by treatment with CD137 monoclonal antibody Clone 2 A), sh-NAC1 + oe-NC group (mice initially treated with NAC1 knockdown lentivirus and overexpression lentivirus control for 3 days, followed by treatment with CD137 monoclonal antibody Clone 2 A), and sh-NAC1 + oe-LDHA group (mice initially treated with NAC1 knockdown lentivirus and LDHA overexpression lentivirus for 3 days, followed by treatment with CD137 monoclonal antibody Clone 2 A). The specific knockdown sequence for sh-NAC1 was as follows: 5’-CCGGGCTGAACTTATCAACCAGATTCTCGAGAATCTGGTTGATAAGTTCAGCTTTTTTG-3’. The lentivirus used had a titer of 1 × 10^11^ PFU and was procured from Gima Biological. The lentivirus was administered via tail vein injection at an approximate volume of 50 μL per mouse, and this administration was carried out twice a week for a total duration of 7 weeks.

At day 21, liver tissue samples were harvested from six mice in each experimental group for comprehensive histological analysis. This analysis encompassed the assessment of pathological changes in liver tissue and collagen fibrosis. Furthermore, liver cell apoptosis within the tissue was examined using TUNEL staining. Additionally, the levels of hepatic fibrosis markers, namely hydroxyproline (ab222941, abcam, UK) and hyaluronic acid (ab2877991, abcam, UK), were quantified in the homogenized mouse liver tissue utilizing specific assay kits. Subsequently, at the 7-month time point, six mice from each group were selected for further evaluation. Liver morphology in these mice was closely observed, and liver nodules were scrutinized through H&E staining. The formula used for calculating liver tumor volume is as follows: tumor volume = ½ L*W^2, where L represents tumor length and W represents tumor width. Additionally, the expression of the CD8 protein within the liver tissue was assessed using IHC staining techniques [28].

### Pathological histology analysis

Liver tissues were collected from each group of mice, and liver sections with a thickness of 5 μm were prepared and embedded in paraffin. Subsequently, these sections were subjected to observation of inflammatory reactions and analysis of liver collagen deposition/accumulation using H&E staining and Sirius Red staining. For H&E staining, the following protocol was followed: Hematoxylin and eosin staining was conducted using PT001 reagent (procured from Shanghai Bogu Biological Technology Co., Ltd., Shanghai, China) at room temperature for a duration of 10 min, followed by washing in running water for 30–60 s. Differentiation was achieved by immersing the sections in 1% hydrochloric acid alcohol for 30 s, followed by another washing step in running water and subsequent immersion in 1% eosin solution at room temperature for 1 min. Dehydration was performed using a gradient of alcohol (with concentrations of 70%, 80%, 90%, 95%, and 100%), with each concentration applied for 1 min. This was followed by clearing with xylene for 1 min. The sections underwent two rounds of transparency treatment with xylene I and xylene II, each lasting 1 min. Finally, the sections were mounted with neutral gum within a fume hood. The ensuing liver tissue morphological changes or structural characteristics were observed and documented using an optical microscope (BX50; Olympus Corp, Tokyo, Japan) [45].

Sirius Red staining: Liver tissue sections were stained with a saturated solution of 0.1% Sirius Red in 0.1% picric acid, and rapidly turned green after 1 h. The distribution of collagen protein was quantified by Image Pro Plus software (Media Cybernetics, Bethesda, MD) to measure the positive staining area of the tissue. Four consecutive images (×40) were taken for each section [38].

### TUNNEL staining

To detect liver cell apoptosis, the TUNEL Apoptosis Detection Kit-DAB (Catalog No. abs50022, ABclonal Biotech Co., Ltd., Shanghai, China) was utilized. The specific procedure is as follows: Liver tissue sections were sliced at room temperature. These sections were then incubated with the TdT/nucleotide complex for a duration of 1 h. Following the incubation, the sections were washed with PBS. Nuclear labeling was performed using horseradish peroxidase and diaminobenzidine (DAB). Subsequently, counterstaining was carried out using Hematoxylin. Apoptotic cells were observed under an optical microscope, and images were captured. Positive apoptotic cells exhibited a brown staining in the nucleus. In each section, 5 randomly selected fields of view were assessed to count and detect apoptotic cells. The apoptosis rate was quantified as a percentage of positive cells, calculated by dividing the number of positive apoptotic cells by the total cell count and multiplying by 100% [46].

### Immunohistochemistry

Following the fixation of liver tissues from each group of mice using formalin, tissue sections were obtained by embedding the tissues in paraffin. After deparaffinization, rehydration, and antigen retrieval through slicing, the tissues were subjected to incubation with primary antibodies against F4/80 (diluted at 1:5000, ab300421, Abcam, UK) and CD8 (diluted at 1:2000, ab217344, Abcam, UK) to assess the expression of the relevant proteins [47]. Subsequently, observations were made, and images were captured using an optical microscope (CX43, Olympus, Japan) to document the expression patterns and distribution of these proteins within the liver tissue sections.

### Western blot

To extract total cellular protein from each experimental group, a protein extraction kit (BB-3101, Bestbio, Shanghai, China) was employed. The protein concentration in each sample was quantified using a BCA protein assay kit (Beyotime Biotechnology, P0012S, China) and adjusted with deionized water as necessary. Subsequently, a 10% SDS-PAGE gel (P0012A, Biomics Biotechnologies Co., Ltd., Shanghai, China) was prepared. Each well was loaded with 50 μg of protein sample, and electrophoresis was conducted at constant voltages of 80 V and 120 V for 2 h, as appropriate. The protein content was then transferred onto a PVDF membrane (ISEQ00010, Millipore, Billerica, MA, USA) using the wet transfer method, applying a constant current of 250 mA for 90 min. Following the transfer, the PVDF membrane was blocked by incubating it in TBST buffer containing 5% skim milk powder for 2 h. After discarding the blocking solution, the membrane was washed once with TBST before being exposed to primary antibodies overnight at 4°C (see Table S2 for antibody details). Subsequently, the membrane was washed three times with TBST for 10 min each. A goat anti-rabbit IgG antibody labeled with horseradish peroxidase (ab6721, Abcam, Cambridge, UK) was applied at a dilution of 1:2000 and incubated at room temperature for 1 h. Following this incubation, the membrane was washed three times with PBST for 10 min each. The membrane was then immersed in an ECL reaction solution (Western blotKLS0100, Millipore) for visualization, and the developed results were obtained by exposing the membrane in a dark box. For quantification, the relative gray value between the target protein band and the reference band, typically β-actin, was calculated, representing the relative expression level of the protein of interest.

### RT-qPCR

Total RNA was extracted from the samples using TRIzol reagent (15596018, Invitrogen, USA). Subsequently, the High-Capacity cDNA Reverse Transcription Kit (4368813, Invitrogen, USA) was employed to reverse transcribe the extracted total RNA into cDNA, utilizing β-actin as the internal reference. For real-time PCR (RT-PCR) experiments, an ABI7500 real-time PCR instrument (Thermo, USA) was utilized in conjunction with the SYBR® Premix Ex TaqTM (Tli RNaseH Plus) kit (RR820A, TaKaRa, Japan). The PCR reaction mixtures underwent amplification using a real-time fluorescence quantitative PCR instrument from ABI, USA. To analyze the final data, the 2-ΔΔCt method was employed. The primer sequences utilized in these experiments were synthesized by Invitrogen and are detailed in Table S3.

### Statistical analysis

All data analyses were conducted using SPSS 21.0 statistical software (IBM, USA). Continuous variables were expressed as mean ± standard deviation. The *t*-test was employed to compare data between two groups, whereas one-way analysis of variance (ANOVA) was utilized for comparisons involving multiple groups. A significance level of *P* < 0.05 was considered to indicate statistically significant differences.

### Supplementary information

Supplementary information

## Data Availability

The original contributions presented in the study are included in the article/supplementary materials, further inquiries can be directed to the corresponding author/s.
