# Supplementary material for: NAC1 transcriptional activation of LDHA induces hepatitis B virus immune evasion leading to cirrhosis and hepatocellular carcinoma development
Source: Oncogenesis. 2024 May 4;13(1):15. doi: 10.1038/s41389-024-00515-4 (PMC11069585; doi:10.1038/s41389-024-00515-4)
Supplement: Supplementary file 1 — Supplementary information [file 41389_2024_515_MOESM1_ESM.pdf]

**Table S1. NaC1 knockdown sequence**

| Name | Sequence (5’-3’) |
|---|---|
| sh-NAC1-1 | 5'-CCGGGCTGAACTTATCAACCAGATTCTCGAGAATCTGGTTGATAAGTTCAGCTTTTTTG-3' |
| sh-NAC1-2 | 5'-CCGGTCCGCTCTTCTACCAACGATCCTCGAGGATCGTTGGTAGAAGAGCGGATTTTTG-3' |

**Table S2. Western blot antibody information**

| Name | Manufacturer | Cat. | Dilution Ratio |
|---|---|---|---|
| NAC1 | Abcam | ab29047 | 1:1000 |
| LDHA | Abcam | ab300637 | 1:1000 |
| α-SMA | Abcam | ab232784 | 1:1000 |
| COL1A1 | Abcam | ab 260043 | 1:1000 |
| MMP2 | Abcam | ab92536 | 1:1000 |
| PCNA | Abcam | ab92552 | 1:1000 |
| β-actin | Abcam | ab5694 | 1:1000 |

**Table S3. RT qPCR primer sequences**

| Gene | Sequence（5’-3’） |
| --- | --- |
| NAC1（human） | F：GGCCTGGCCCACAATGAA |
| | R： TGACACGTCACAGTACAGGC |
| β-actin（human） | F：ACAGAGCCTCGCCTTTGCC |
| | R：TGGGGTACTTCAGGGTGAGG |

F. Forward; R. Reverse.
